# The diversity, community dynamics, and interactions of the microbiome in the world’s deepest blue hole: insights into extreme environmental response patterns and tolerance of marine microorganisms

**DOI:** 10.1128/spectrum.00531-23

**Published:** 2023-10-20

**Authors:** Biao Chen, Kefu Yu, Liang Fu, Yuxin Wei, Jiayuan Liang, Zhiheng Liao, Zhenjun Qin, Xiaopeng Yu, Chuanqi Deng, Minwei Han, Honglin Ma

**Affiliations:** 1 Guangxi Laboratory on the Study of Coral Reefs in the South China Sea, Coral Reef Research Center of China, School of Marine Sciences, Guangxi University, Nanning, China; 2 Southern Marine Science and Engineering Guangdong Laboratory (Zhuhai), Zhuhai, China; 3 Sansha Track Ocean Coral Reef Conservation Research Institute Co. Ltd., Qionghai, China; 4 Key Laboratory of Environmental Change and Resource Use in Beibu Gulf, Ministry of Education, Nanning Normal University, Nanning, China; Institut Ruder Boskovic, Zagreb, Croatia

**Keywords:** microbiome community dynamics, response pattern, tolerance threshold, extreme environment, deepest blue hole

## Abstract

Blue holes are unique marine sinkholes with extreme environments and biogeochemical processes. However, our understanding of community dynamics, functional profiles, and microbial interactions in blue holes remains limited. We studied the extreme environmental response pattern of the microbiome (Symbiodiniaceae, bacteria, archaea, and fungi) across 14 depths in the world’s deepest blue hole, the Sansha Yongle Blue Hole. The α-diversities of Symbiodiniaceae and archaea were stable to extreme environmental conditions, whereas those of bacteria and fungi varied. Physical and nutrient factors primarily influenced the β-diversities of these four microbes, and there were significant differences in microbial communities among water layers. Nine microbial taxa of *Cladocopium* sp, γ-proteobacteria, Nanoarchaeota, and Ascomycota representing the core microbiome occurred in all water layers. These four microbial groups exhibited potential interactions, with a positive correlation between Symbiodiniaceae and archaea α-diversities. The microbial biogeochemical profiles exhibited notable enrichment characteristics among distinct water layers. Archaea metabolized sulfides in the oxic and upper deep layers, while bacteria dominated sulfide decomposition in the chemocline and lower deep layers. These findings suggest the acclimation of Symbiodiniaceae to an extreme environment may rely on archaea, as a result of a partial niche overlap. The bacterial communities exhibited an environmental response pattern consistent with the Anna Karenina effects, whereas fungal communities displayed an opposite trend. The wide tolerance of the core microbiome to environmental gradients may be linked to evolution, acclimatization, and symbiosis. Bacteria, archaea, and fungi have distinct ecological niches and biogeochemical functions in the Sansha Yongle Blue Hole.

**IMPORTANCE:**

This study comprehensively examined the community dynamics, functional profiles, and interactions of the microbiome in the world’s deepest blue hole. The findings revealed a positive correlation between the α-diversities of Symbiodiniaceae and archaea, indicating the potential reliance of Symbiodiniaceae on archaea in an extreme environment resulting from a partial niche overlap. The negative association between the α-diversity and β-diversity of the bacterial community suggested that the change rule of the bacterial community was consistent with the Anna Karenina effects. The core microbiome comprised nine microbial taxa, highlighting their remarkable tolerance and adaptability to sharp environmental gradient variations. Bacteria and archaea played significant roles in carbon, nitrogen, and sulfur cycles, while fungi contributed to carbon metabolism. This study advanced our understanding of the community dynamics, response patterns, and resilience of microorganisms populating the world’s deepest blue hole, thereby facilitating further ecological and evolutional exploration of microbiomes in diverse extreme environments.

## INTRODUCTION

Blue holes belong to the ecosystem of anchialine caves or cenotes, which exhibit a deep blue-colored, water-filled, vertical karst opening in carbonate rocks (1). Numerous blue holes were formed approximately 2.5 million years ago during the Quaternary period owing to a rise in global sea levels and the dissolution or collapse of carbonate rocks (2 – 5). Therefore, blue holes are potential time capsules that contain records of archive regional and global environmental events (1, 6 – 12). Biogeochemical cycles, hydrologic conditions, and biocoenotic dynamics of blue holes have been explored worldwide (5, 13, 14). Many blue hole ecosystems contain multiple pycnoclines (15), and the environmental, geochemical, and ecological characteristics of these blue holes are similar to those of extreme environments. Generally, blue holes have unique geomorphological characteristics, and water exchange and vertical mixing are limited, leading to transitions from aerobic to anaerobic conditions occurring within a few dozen to several hundred meters without impacting the external environments (16 – 18). Vertical decline of photosynthesis and accumulation of sulfides result in the formation of extreme environmental conditions in the intermediate and deep-water layers of a blue hole (7, 16, 19). Notably, low oxygen availability, less light, and high sulfide concentrations in these blue hole ecosystems were similar to those of deep-sea hydrothermal vents (20 – 23), dark ocean regions (24, 25), hypersaline environments (26), and terrestrial geothermal springs (27), all of which encompass recognized extreme environments (28). Thus, the habitat of blue holes provides rapidly evolving and diverse ecological niches with an abundance of microorganisms. The Bahamas blue hole, characterized by anoxic and microoxic conditions, exhibits a high abundance of the anoxygenic phototroph clade, Chlorobi, and a low presence of Deltaproteobacteria. These major clades harbor distinct microbial biomass and species compositions, as demonstrated by substantial differences (29). Additionally, the Hospital hole also includes chemocline and sulfidic anoxic layers. The unique functions and interaction of the microbial community have notable differences in each layer, and there is evidence of syntrophic relationships between methane oxidizers, methanogens, and sulfate reducers (14). The Shark Bay blue hole (30), Lucayan Cavern (31), Bjejajka Cave (32), Lenga Pit (32), and Vanuatu anchialine cave (33) have also been explored, revealing a high level of phylogenetic diversity, distinct community structure, and diverse ecological functions of microbes (5, 13, 30). Therefore, enclosed offshore blue holes can be used to investigate microbial community dynamics, extreme environmental adaptability, and biogeochemical contributions under natural conditions.

**Fig 1:**
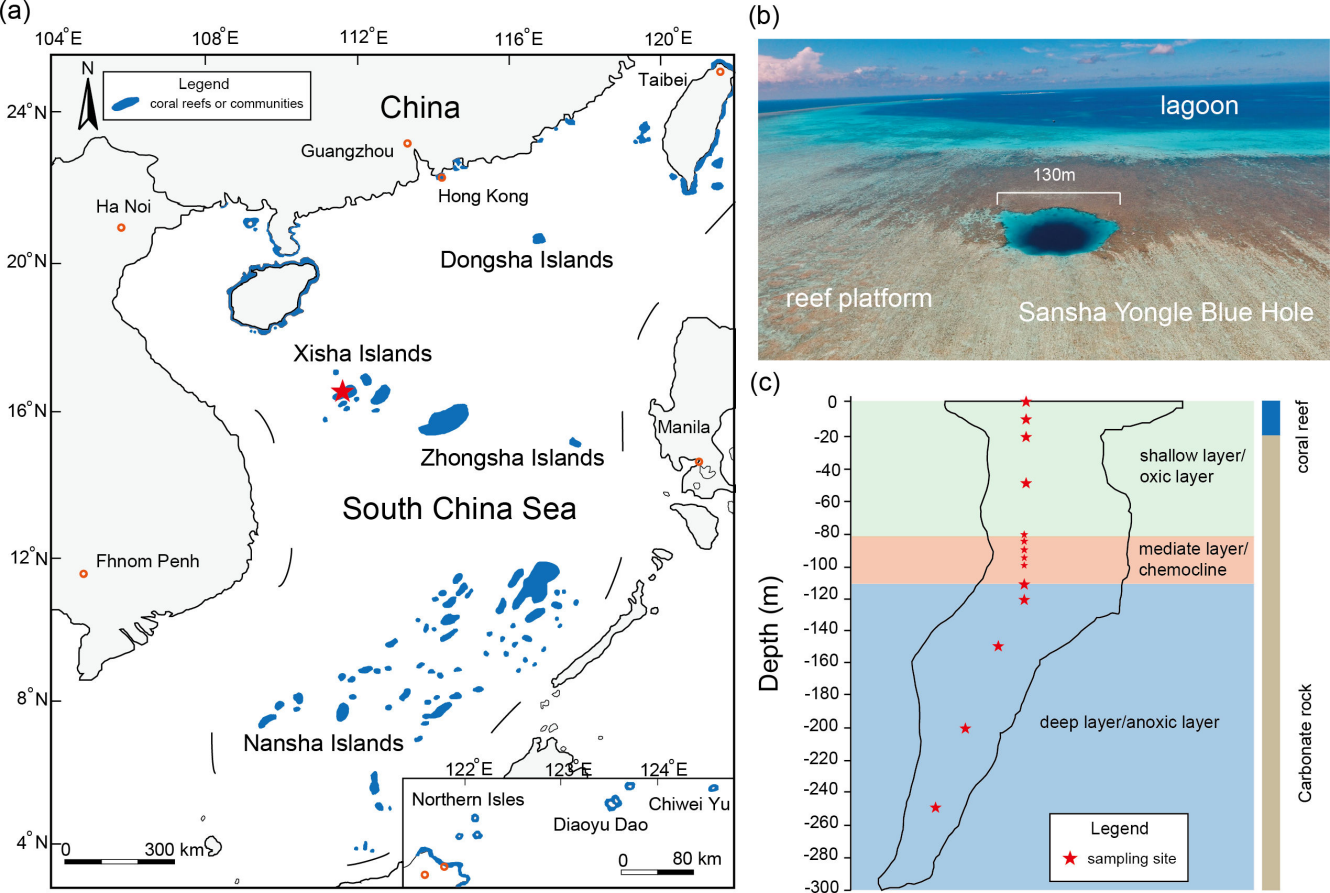
The location of the Sansha Yongle Blue Hole in the South China Sea. (**a**) The SYBH located in Xisha Islands, (**b**) which developed in reef platform of Yongle atoll. (**c**) The sampling depths of the SYBH. The map in panel a is republished from *Frontiers in Microbiology* (65).

The Sansha Yongle Blue Hole (SYBH), which is a water-filled karst opening in the intertidal reef platform of the eastern Yongle atoll in the South China Sea (34, 35), is the deepest (301.19 m) blue hole in the world (Fig. 1a and b). The surface coral reef (0–17 m) and carbonate rock (17–301.19 m) constitute the SYBH, which was formed in 31–29 kyr before present (BP) (35). The SYBH is an enclosed vertical ecosystem, and its water column is strongly stratified and can be divided into oxic (0–80 m), chemocline (80–115 m), and deep anoxic water layers (115–301.19 m; Fig. 1c) (36). Thus, SYBH provides a natural laboratory to determine extreme environmental response patterns and tolerance of reef and other marine microorganisms. The Dinophyceae dominated in eukaryotic microalgae community in the SYBH (37), the abundance of which can most likely be attributed to the contribution of Symbiodiniaceae that can be found in coral reef water and are acquired by coral horizontal transmission (38 – 41). Symbiodiniaceae are distributed in tropical, subtropical, and temperate oceans and exhibit wide environmental tolerance thresholds, diverse symbioses, and ecological functions (42 – 44). Symbiodiniaceae also exhibit strong mutualistic or epiphytic abilities and have the potential to interact with marine microbes (40, 43, 44). Symbiodiniaceae established numerous interactions with bacteria, and the increase in their α-diversity can lead to a decline in the complexity interaction network of the microbial community (45). Even pure culture strains of Symbiodiniaceae harbor specific bacteria taxa and engage in interactions (46 – 48). Although the response patterns of Symbiodiniaceae to variations in temperature (41, 45, 49 – 51), pH (52, 53), salinity (54 – 56), and light intensity (57) have been determined, their adaptive and microbial interaction characteristics in extreme environments with low light, low oxygen availability, and high sulfide concentrations below 50 depths in SYBH remain unknown (36). Additionally, previous studies have focused on the development and evolution (35, 58) and the hydrochemical properties (36) and diversities of bacteria and archaea, such as potential animal and plant pathogenic bacteria (*Vibrio*) (59 – 62), distribution of benthic foraminifera and other eukaryotes (37, 63), and biogeochemical cycles (carbon cycling) (34) of the SYBH, and suggested that diverse microorganisms may play a key role in the ecological function of water columns. Nevertheless, it remains unclear how the core microbiome, changing roles, interactions, and tolerance limits are associated with the ability of microbial communities to respond to extreme environments and the gradient variations in SYBH. Moreover, fungi are phylogenetically and functionally diverse ubiquitous components of ocean ecosystems, but the diversity, community structure, functional profiles, and environmental adaptability of this key ecologically functional group remain unclear (64).

This study aimed to comprehensively examine the diversity variations, community dynamics, core taxa, interactions, and ecological functions of the microbiome among the oxic, chemocline, and anoxic water layers across 14 depths in the SYBH; this will enhance our comprehension of the tolerance threshold and adaptability of marine microorganisms in response to extreme environments characterized by low oxygen availability, weak light, and high sulfide concentrations.

## RESULTS

### The community composition of microbiome

**Fig 2:**
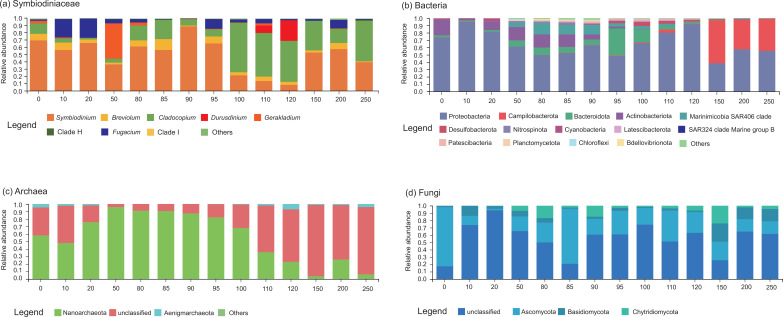
The community composition of microbiome in Sansha Yongle Blue Hole. The taxonomic profile of the abundant communities of (**a**) Symbiodiniaceae, (**b**) bacteria, (**c**) archaea, and (**d**) fungi in distinct water layers (0–250 m) in the SYBH.

**Fig 3:**
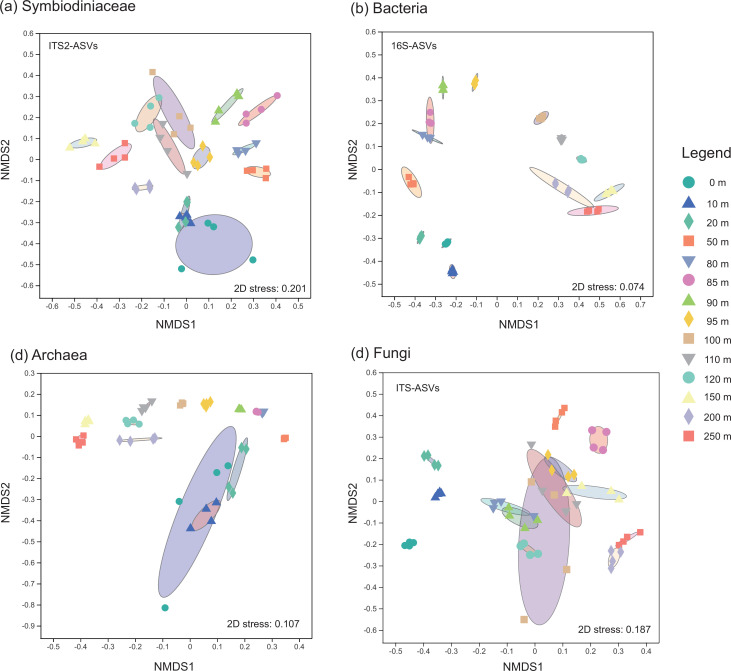
The microbiome community structure and relative dispersion in the Sansha Yongle Blue Hole. The non-metric multidimensional scaling (NMDS) of Bray-Curtis distances of the compositions of (**a**) Symbiodiniaceae-, (**b**) bacteria-, (**c**) archaea-, and (**d**) fungi-associated seawater samples in distinct depths in (YBH).

Based on the local alignment of internal transcribed spacer region 2 (*ITS2*) reads, all genera and subclades of Symbiodiniaceae, except free-living *Effrenium*, were identified. The Symbiodiniaceae community was dominated by *Symbiodinium* (50.4% ± 23.8%), *Cladocopium* (26.2% ± 22.9%), *Fugacium* (8.1% ± 8.8%), and *Breviolum* (6.9% ± 5.7%) in the SYBH (Fig. 2a). *Gerakladium* (4.7% ± 13.3%) dominated the Symbiodiniaceae community at the 50 m depth (48.9%), while *Durusdinium* (3.1% ± 8.2%) had the highest relative abundance at the 110–120 m depth (110 m: 9.9%; 120 m: 28.6%). Regarding the bacterial community, 45 phyla, 104 classes, 247 orders, 397 families, 686 genera, 1,090 presumptive species, and 3,229 amplicon sequence variants (ASVs) were identified. Proteobacteria (66.6% ± 17.4%), Campilobacterota (10.7% ± 20.2%), Bacteroidetes (7.3% ± 9.9%), and Actinobacteria (7.0% ± 8.3%) dominated the bacterial community in the SYBH (Fig. 2b). Proteobacteria were widely distributed in the SYBH and dominated the bacterial communities in all water layers. Actinobacteriota and Bacteroidota showed high relative abundance at depths of 0–95 m (12.2% ± 7.5%) and 0–120 m (9.2% ± 10.4%) in the shallow water layers of the SYBH, respectively, while Campilobacterota dominated the bacterial communities in deep-water layers (150–250 m, 47.4% ± 9.9%). Sulfide-consuming Desulfobacterota were mainly distributed in the intermediate and deep layers from 90 to 120 m (3.3% ± 1.9%), but Nitrospinota were highly abundant in the shallow and intermediate layers at 50–90 m (2.1% ± 0.9%). A total of 6 phyla, 9 classes, 10 orders, 11 families, 11 genera, 14 presumptive species, and 3,797 ASVs of archaea were found in the SYBH. Nanoarchaeota and Agenigamarchaeota dominated the archaeal community (Fig. 2c), which were all aligned with the DPANN superphyla. Nanoarchaeota were almost ubiquitous in the SYBH water (56.5% ± 32.7%). However, Agenigamarchaeota mainly lived in shallow (0–20 m; 2.9% ± 1.5%) and deep water (110–250 m; 3.2% ± 2.5%) regions. Numerous unclassified archaeal phylotypes were distributed, which had a high relative abundance in all water layers (41.7% ± 31.4%), especially in the deep-water area (150–250 m; 85.8% ± 11.6%). Regarding fungi, 4 phyla, 12 classes, 20 orders, 27 families, 27 genera, 30 presumptive species, and 7,888 ASVs were identified in the SYBH. The fungal community was dominated by Ascomycota, Basidiomycota, and Chytridiomycota (Fig. 2d). Ascomycota (30.2% ± 22.2%) and Basidiomycota (7.7% ± 7.3%) were widely distributed in the SYBH, but a high abundance of Chytridiomycota was identified in the water layers of 50–250 m depth (7.9% ± 7.4%). Similar to the archaeal community, unclassified fungal phylotypes were widely distributed and had a high relative abundance in all water layers of the SYBH (55.9% ± 21.6%). There were significant differences between the community compositions of Symbiodiniaceae [permutation multifactorial analysis of variance (PERMANOVA): *R* ^2^ = 0.6926; *P* = 0.0001], bacteria (PERMANOVA: *R* ^2^ = 0.9958; *P* = 0.0001), archaea (PERMANOVA: *R* ^2^ = 0.8547; *P* = 0.0001), and fungi (PERMANOVA: *R* ^2^ = 0.7661; *P* = 0.0001) in the distinct water layers of the SYBH (Fig. 3), indicating that the microbial community composition showed high flexibility and variability.

### The changing rules of α-diversity and β-diversity of microbiome

**Fig 4:**
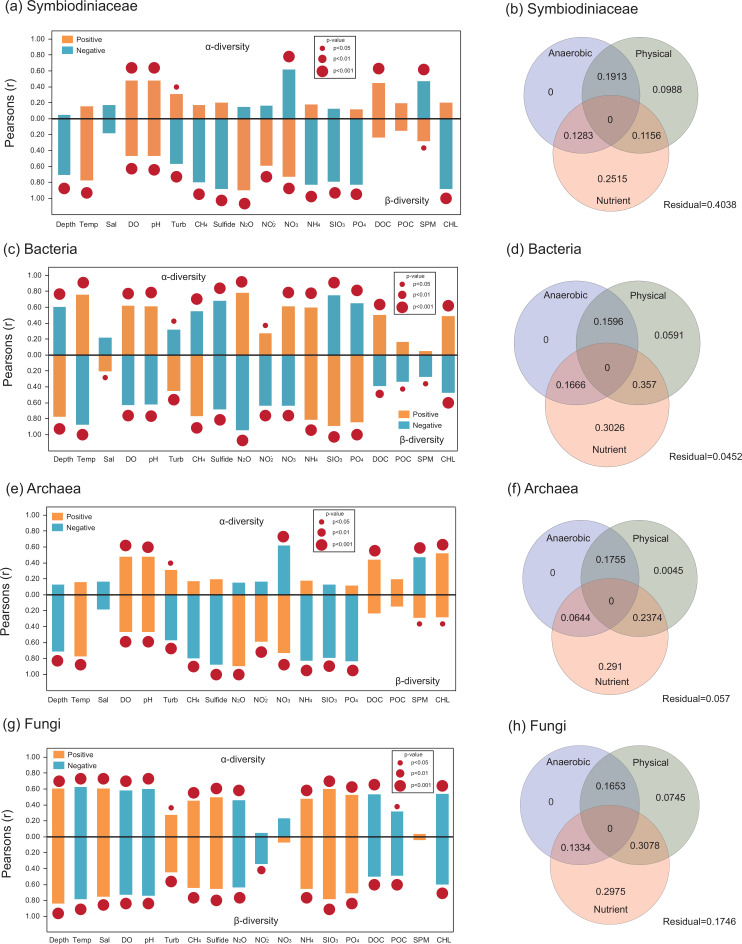
Environmental drivers of α-diversity and β-diversity for microbiome in the Sansha Yongle Blue Hole. Positive (orange) and negative (blue) Pearson’s correlation results comparing α-diversity and β-diversity of (**a**) Symbiodiniaceae, (**c**) bacteria, (**e**) archaea, and (**g**) fungi with distinct anaerobic (CH_4_, sulfide, N_2_O), physical [depth (**m**) temperature (Temp: °C), salinity (Sal: PSU), DO (mg/L), pH, Turb (FNU)], and nutrient [NO_3_^−^ (μmol/L), NO_2_^−^ (μmol/L), NH_4_^+^(μmol/L), SiO_3_^2−^ (μmol/L), PO_4_^3−^ (μmol/L), DOC (μmol/L), POC (μmol/L), SPM (mg/L), Chl *a* (μg/L)] parameters at the SYBH. (**b, d, f, h**) The variation partitioning analysis of relative contribution of anaerobic, physical, and nutrient parameters in microbial β-diversity.

The α-diversity of Symbiodiniaceae was influenced by diverse environmental factors in the SYBH (Table 1; Fig. 4a). Among the physical factors, only pH, concentration of dissolved oxygen (DO), and turbidity (Turb) showed a positive association with the Shannon H’ of Symbiodiniaceae, while the anaerobic products of methane (CH_4_), sulfide, and nitrous oxide (N_2_O) were not associated with the α-diversity of Symbiodiniaceae. The Shannon H’ index value of Symbiodiniaceae was unaffected by all nutritive factors. The concentration of dissolved organic carbon (DOC) showed a significantly positive correlation with the α-diversity of Symbiodiniaceae, while there were no obvious associations between nitrate (NO_3_^−^) and suspended particulate matter (SPM) and the Shannon H’ of Symbiodiniaceae (Fig. 4a). The β-diversity of Symbiodiniaceae showed high flexibility in response to diverse environmental factors. Physical, anaerobic, and nutrient factors, except for salinity, DOC, and particulate organic carbon (POC), were significantly associated with the β-diversity of Symbiodiniaceae in the SYBH. Temperature, DO, pH, and concentrations of N_2_O, nitrite (NO_2_^−^), NO_3_^−^, and SPM increased the dispersal degree of the Symbiodiniaceae community, while the β-diversity of Symbiodiniaceae showed a significantly negative correlation with depth, Turb, CH_4_, sulfide, ammonium (NH_4_^+^), silicate (SiO_3_^2-^), phosphate (PO_4_^3-^), and chlorophyll *a* (Chl *a*). Variation partitioning analysis (VPA) showed that the independent effects of nutrient products were the largest contributor to the variation in Symbiodiniaceae community structure (25.15%) (Fig. 4b).

**TABLE 1:** Summary of the environmental factors across 14 depths among three water layers in the Sansha Yongle Blue Hole

Water layers	Depth (m)	Temperature (℃)	Salinity (PSU)	DO (mg/L)	pH	Turbidity (FNU)	Chlorophyll *a* (μg/L)	SPM (mg/L)	NO_3_^−^(μmol/L)	NO_2_^−^(μmol/L)	NH_4_^+^ (μmol/L)	SIO_3_^2−^(μmol/L)	PO_4_^3−^(μmol/L)
Oxic layer	0	28.3	33.45	6.95	8.3	0.61	0.194	13.451	0.000	0.084	0.000	0.000	0.000
	10	28.1	33.6	6.83	8.29	0.6	0.119	4.158	0.000	0.095	0.000	3.459	0.000
	20	28.2	33.59	6.8	8.29	0.64	0.169	3.913	1.217	0.243	0.000	3.027	0.065
	50	28.2	33.12	6.85	8.29	0.62	0.158	4.158	4.660	0.097	0.000	5.622	0.196
Chemocline	80	23.1	34.09	1.03	7.77	0.28	0.033	7.337	5.321	0.095	0.000	36.757	0.348
	85	22.5	34.17	0.57	7.74	0.32	0.032	20.299	4.679	0.145	2.609	51.892	0.630
	90	21.7	34.27	0.25	7.74	0.38	0.025	33.016	0.875	0.232	5.217	65.730	0.935
	95	20.8	34.41	0.02	7.76	0.96	0.062	23.478	0.438	0.204	11.739	80.432	1.065
	100	19.8	34.55	0.01	7.76	0.38	0.035	10.272	0.000	0.063	18.587	86.487	1.196
	110	18.6	34.5	0.01	7.76	0.28	0.024	9.783	0.000	0.063	22.500	92.108	1.717
Anoxic layer	120	17.9	34.49	0.01	7.72	0.34	0.023	8.804	0.000	0.061	25.761	98.162	2.261
	150	15.6	34.52	0	7.7	1.01	0.055	5.625	0.000	0.076	99.783	132.324	5.065
	200	15.3	34.56	0	7.71	1.38	0.065	3.424	0.000	0.059	100.109	133.622	5.370
	250	14.5	34.59	0	7.69	1.54	0.065	7.337	0.000	0.019	103.696	134.054	5.152

Regarding the bacterial community, both α- and β-diversity showed high flexibility in response to environmental influences. Although temperature, DO, pH, N_2_O, NO_2_^−^, NO_3_^−^, NH_4_^+^, DOC, and Chl *a* showed significantly positive correlations with the Shannon H’ of bacteria, the increase in depth, Turb, and the concentration of CH_4_, sulfide, SiO_3_^2−^, and PO_4_^3−^ decreased the α-diversity of bacteria in the SYBH (Fig. 4c). Additionally, the changes in α- and β-diversity were notably different under environmental effects, except for that of NH_4_^+^ and sulfide. The increase in α-diversity accompanied a decrease in the dissimilarity of the bacterial community. Physical, anaerobic, and nutrient factors explained 95.48% of the changes in the bacterial community in seawater from the SYBH, and the degree of explanation was the highest in the microbial community in the SYBH (Fig. 4d). The combined effect of physical and nutrient factors contributed to the highest percentage of variation (35.70%) in the community structure of bacteria in the SYBH.

The changes in α- and β-diversities of archaea were similar to those of Symbiodiniaceae in the SYBH. Correlations between Shannon H’ of archaea and environmental factors were weak, except for DO and pH among physical factors and NO_3_^−^, DOC, SPM, and Chl *a* among nutritional factors (Fig. 4e). The α-diversity of archaea was not affected by changes in CH_4_, sulfide, and N_2_O concentrations. Correlations between the environmental factors and β-diversity of the archaeal community were consistent with those of Symbiodiniaceae, except for the concentration of Chl *a*. The degree of dispersion of the archaeal community was positively associated with temperature, DO, pH, and the concentrations of N_2_O, NO_2_^−^, NO_3_^−^, SPM, and Chl *a*, whereas an increase in depth, Turb, and the concentrations of CH_4_, sulfide, NH_4_^+^, SiO_3_^2−^, and PO_4_^3−^ decreased the β-diversity of archaea in the SYBH. VPA also showed that the independent effect of nutrient factors accounted for the highest percentage of variation in the archaeal community in the SYBH (29.1%; Fig. 4f).

At the per-sample level, the changing rules in α- and β-diversity of fungi in the SYBH were highly correlated (Fig. 4g), indicating that an increase in α-diversity would decrease the stability of the fungal community. The α- and β-diversity of fungi showed high flexibility in responding to changes in physical, anaerobic, and nutrient factors in the SYBH. In addition to NO_2_^−^, NO_3_^−^, and SPM, other environmental factors also affected the Shannon H’ of fungi. Moreover, the β-diversity of fungi was not significantly associated with the concentrations of NO^3−^ and SPM, which were affected by other environmental factors. VPA showed that the combined effect of physical and nutrient products made the highest contribution (30.78%) to the variation in fungal community structure in the SYBH (Fig. 4h).

### The core microbiome

**Fig 5:**
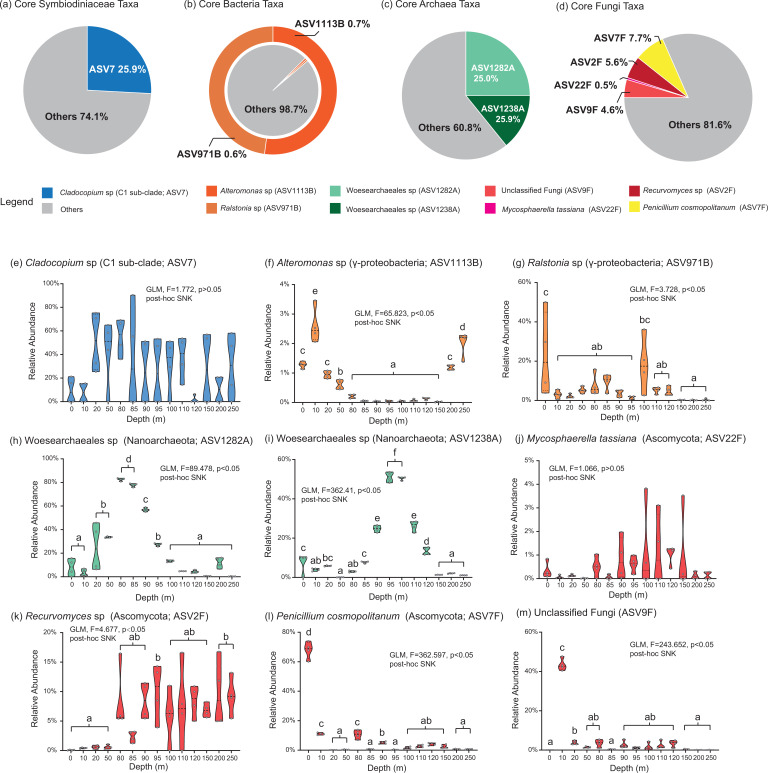
The community composition and change trend of core microbiome in the Sansha Yongle Blue Hole. (**a–d**) The pie chart on the top shows the percentage of core microbiome in microbial community composition. (**e–m**) The change trend of the relative abundance of core microbial ASV in SYBH.

The *Cladocopium* C1 subclade (ASV7; 25.9%) was the dominant core taxon in the Symbiodiniaceae community of the SYBH (Fig. 5a). However, there were no significant differences between the relative abundances of C1 subclade in the Symbiodiniaceae communities at different depths [general linear model (GLM): *F* = 1.772; *P* > 0.05; Fig. 5e]. Two core bacterial taxa, *Alteromonas* sp. (ASV1113B) and *Ralstonia* sp. (ASV971B), were consistently present across all water layers in the SYBH. The core bacterial microbiome, which comprised rare bacterial members in the SYBH, accounted for only 1.3% of the total bacterial abundance (Fig. 5b). Nevertheless, the relative abundances of these core bacteria varied substantially across different depths. *Alteromonas* sp. had the highest relative abundance in the 10-m aqueous layer (GLM: *F* = 65.823; *P* < 0.05; Fig. 5f), whereas the relative abundance of *Ralstonia* sp. on the sea surface (0 m) was significantly higher than that at other depths (GLM: *F* = 3.728; *P* < 0.05; Fig. 5g). Core archaea taxa were dominant members of the archaeal community in the SYBH, with ASV1282A and ASV1238A accounting for 25.0% and 25.9% of the archaeal abundance, respectively (Fig. 5c). All members of the core archaeal microbiome were aligned with the unclassified Woesearchaeales, which belongs to phylum Nanoarchaeota. Regarding the dynamics of core archaea, the relative abundance of ASV1282A sharply increased from 3.4% ± 2.9% (10 m) to 82.6% ± 1.2% (80 m) above 80–85 m. However, it gradually declined from 56.9% ± 1.6% (90 m) to 0.3% ± 0.1% (250 m) in the SYBH (GLM: *F* = 89.478; *P* < 0.05; Fig. 5h). The trend of variation in ASV1238A abundance was similar to that of ASV1282A, which increased sharply, starting from the sea surface (0 m) to a depth of 85 m and reaching a maximum (51.0% ± 2.5%; 95 m) at a depth of 95–100 m. However, the relative abundance of ASV1238A sharply decreased from 26.2% ± 2.6% at 110 m to 1.1% ± 0.1% (250 m) below 110 m (GLM; *F* = 362.41; *P* < 0.05; Fig. 5i). Interestingly, there were four core fungi taxa present at all SYBH depths. Excluding *Mycosphaerella tassiana* (ASV22F; 0.5%), the remaining core fungal taxa dominated in the fungal community (4.6%–7.7%; Fig. 5d). The relative abundance of *Mycosphaerella tassiana* was homogeneous across distinct water layers of the SYBH (GLM: *F* = 1.066; *P* > 0.05; Fig. 5j). The abundance of *Recurvomyces* sp. (ASV2F) in the deep-water area (95, 200, and 250 m) was significantly higher than that in the shallow water regions of the SYBH (0–50 m; GLM: *F* = 4.677; *P* < 0.05; Fig. 5k). Conversely, *Penicillium cosmopolitanum* (ASV7F) and unclassified fungi (ASV9F) exhibited similar patterns of variation, with low relative abundances observed in the dark deep-water area of the SYBH. The relative abundance of *P. cosmopolitanum* reached its maximal value at a depth of 0 m from the sea surface, sharply declining from 67.9% ± 5.8% at 0 m to 0.6%–11.1% below 10 m and then remaining relatively constant until the bottom of the SYBH (GLM: *F* = 362.597; *P* < 0.05; Fig. 5l). The relative abundance of ASV9F also peaked at a depth of 10 m and was significantly higher than that in other water layers of the SYBH (GLM: *F* = 243.652; *P* < 0.05; Fig. 5m).

### The enrichment characteristics of ecological functions of microbiome

**Fig 6:**
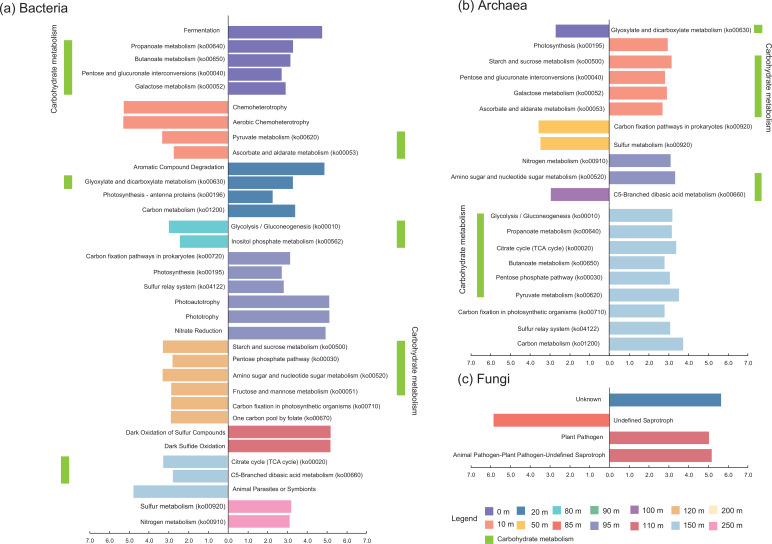
Enrichment characteristics of functional traits in microbiome from the Sansha Yongle Blue Hole. Enrichment functional traits with LDA scores of 2 or greater in microbial communities of (**a**) bacteria, (**b**) archaea, and (**c**) fungi among different water layers. The functional profiling predicting of bacteria was used to PICRUST 2 and FAPROTAX, and those of archaea only was used to PICRUST 2. The function of fungi communities was predicted by FUNGuild.

The bacterial community was enriched in chemoheterotrophy and aerobic chemoheterotrophy functions at 10 m in the SYBH [linear discriminant analysis (LDA): 5.6–5.7; *P* < 0.05; Fig. 6a] but photoautotrophy and phototrophy at 20 and 95 m (LDA: 2.5–5.3; *P* < 0.05; Fig. 6a). Nitrate metabolism in the bacterial community was enriched at 95 and 250 m (LDA: 3.7–5.0; *P* < 0.05; Fig. 6a) and sulfur metabolism at 95, 110, and 250 m (LDA: 3.6–5.4; *P* < 0.05; Fig. 6a). Bacterial functional profiles of fixed or metabolized carbon were highly abundant at 20, 95, and 120 m (LDA: 3.7–4.4; *P* < 0.05; Fig. 6a) and carbohydrate metabolism at 0, 10, 20, 80, 120, and 150 m (LDA: 2.3–3.2; *P* < 0.05; Fig. 6a). In the archaeal community, photosynthesis was enriched at 10 m (LDA: 2.9; *P* < 0.05; Fig. 6b). Regarding the nitrogen cycling processes, only one enriched functional profile (nitrogen metabolism; ko00910) was observed in the archaeal community at 95 m (LDA: 3.0; *P* < 0.05; Fig. 6b). The archaeal community had a high abundance of sulfur metabolism traits at 50 m and 150 m (LDA: 2.9–3.3; *P* < 0.05; Fig. 6b), which differed from that of the bacterial community in the SYBH. Carbon metabolism and fixation were also enriched in the archaeal community at 50 m and 150 m (LDA: 3.8–4.2; *P* < 0.05; Fig. 6b). Similar to the bacterial community, the archaeal community was also enriched in carbohydrate metabolism at 0 m, 10 m, 50 m, 95 m, 100 m, and 150 m (LDA: 2.6–3.4; *P* < 0.05; Fig. 6b). FUNGuild analysis showed that the fungal community at 85 m was mainly colonized by undefined saprotrophic fungi (LDA: 5.8; *P* < 0.05; Fig. 6c) and plant pathogens and animal pathogen-plant pathogen-undefined saprotrophic fungi were enriched in the fungal community at 110 m (LDA: 5.1–5.2; *P* < 0.05; Fig. 6c). Fungi with unknown functions were dominant in the 20-m fungal community in the SYBH (LDA: 6.0; *P* < 0.05; Fig. 6c).

### The microbial interaction and network structure

**Fig 7:**
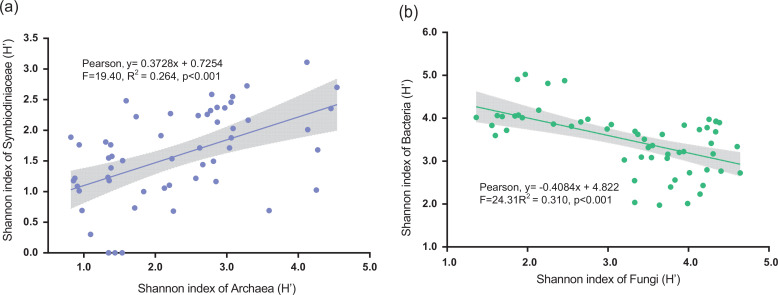
The correlations of alpha diversity among microorganism in the Sansha Yongle Blue Hole. (**a**) Relationship of Shannon index (H′) between archaea and Symbiodiniaceae in the SYBH. (**b**) Correlation of Shannon index (H′) between fungi and bacteria in the SYBH.

The results of the Shannon index correlation analysis indicated that there was a significant positive correlation between the α-diversities of archaea and Symbiodiniaceae (Pearson, *R* ^2^ = 0.264, *P* < 0.001; Fig. 7a; Table 2), while the α-diversities of bacteria and fungi were not associated with those of archaea and bacteria in the SYBH. Shannon H’ index of bacteria showed a significantly negative correlation with that of fungi (Pearson, *R* ^2^ = 0.310, *P* < 0.001; Fig. 7b; Table 2). Partial correlation analysis showed that variation in the α-diversities of bacteria and fungi did not affect the positive correlation between the α-diversities of archaea and Symbiodiniaceae (Pearson, *R* ^2^ = 0.251, *P* < 0.001; Table 2); the negative correlations between bacteria and fungi were also unaffected by changes in the α-diversities of bacteria and Symbiodiniaceae (Pearson, *R* ^2^ = 0.346, *P* < 0.001; Table 2).

**TABLE 2:** Correlation and partial correlation statistics among Symbiodiniaceae, bacteria, archaea, and Fungi

Microbial taxa		Symbiodiniaceae	Bacteria	Archaea	Fungi
Symbiodiniaceae	*r*	–	–	–	–
	*R* ^2^	–	–	–	–
	*P*	–	–	–	–
	*N*	–	–	–	–
Bacteria	*r*	0.084	–	–	–
	*R* ^2^	0.007	–	–	–
	*P*	0.54	–	–	–
	*N*	56	–	–	–
Archaea	*r*	**0.514/0.501^*a*^**	0.211	–	–
	*R* ^2^	**0.264/0.251^*a*^**	0.049	–	–
	*P*	**>0.001/>0.001^*a*^**	0.119	–	–
	*N*	56	56	–	–
Fungi	*r*	0.42	**0.557/0.588^*a*^**	0.74	–
	*R* ^2^	0.176	**0.310/0.346^*a*^**	0.548	–
	*P*	0.758	**>0.001/>0.001^*a*^**	0.588	–
	*N*	56	56	56	–

^*a*^ Bold indicates statistical significance after Pearson correlation test, and adjust r, *R* ^2_,_^ and *P* after partial correlation correction marked by asterisk. "-" means no data.

**Fig 8:**
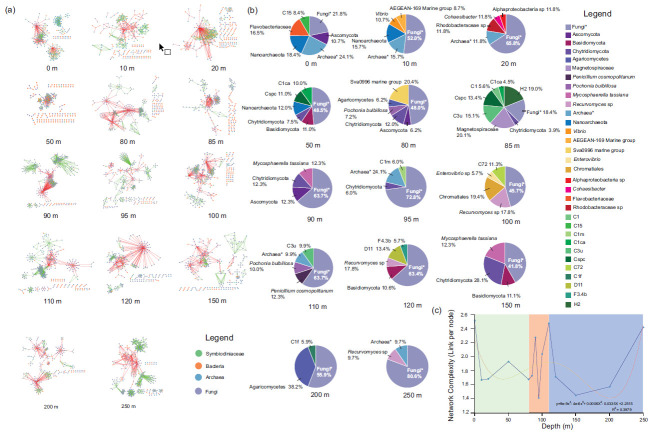
The microbiota interaction network and complexity of microbial community in Sansha Yongle Blue Hole. (**a**) The microbial interaction network in different depths in SYBH. (**b**) The key microbial drivers of interaction network in the SYBH. (**c**) The changing rule of microbial network complexity in the SYBH.

The outcome of the SYBH ecosystem processes is governed by a complex network of direct and indirect interactions between microorganisms. There were abundant interactions among Symbiodiniaceae, bacteria, archaea, and fungi in the SYBH, with all water layers displaying different microbiota interaction networks (Fig. 8). Fungal ASVs dominated the network in all water layers and played the most important role in microbial interactions in the SYBH (Fig. 8a). Fungi also dominated the microbial hub community in all water layers, and many unclassified fungi were key drivers, which showed high relative abundances (21.8%–80.6%) in the microbiota interaction network (Fig. 8b). The members of Ascomycota, Basidiomycota, Chytridiomycota, and Agaricomycetes were key drivers in the microbial interaction network in the SYBH; however, many fungal hubs are often present in extreme natural environments. *Recurvomyces* sp., *Penicillium*, and *Mycosphaerella* were identified in the core microbiome of SYBH. Archaea, as network hubs, played an important role in microbial interactions, a high degree of which was shown by Nanoarchaeota in shallow water layers (0, 10, and 50 m) of the SYBH. Many unclassified archaea hubs were also identified at 0–20, 95 m, 110, and 250 m. The contribution of the active bacterial community to the microbial interaction network in the deep-water layers of the SYBH was limited, with a high abundance of bacteria being identified above the 100-m water column. Flavobacteriaceae (0 m), *Vibrio*, AEGENA-169 marine group, Rhodobacteraceae, Cohaesibacter, and Sva0996 marine groups were key drivers in the shallow microbiome, whereas Magnetospiraceae at 85 m and Chromatiale and *Enterovibrio* at 100 m also acted as microbial network hubs in the intermediate water layers. Symbiodiniaceae, as a key interactional hub, was widely distributed in the SYBH, with the highest total degree in the 85-m water layer. *Cladocopium* had a high degree at 0 (C15), 50 (C1ca, Cspc), 85 (C3u, Cspc, C1, C1ca), 95 (C1m), 100 (C72), 110 (C3u), and 200 m (C1f), whereas *Durusdinium* (D11) as a microbial hub was only identified at 120 m. *Fugacium* and H2 were the key drivers of microbial communities at 85 and 120 m, respectively. Moreover, the index of microbial interaction network complexity in the SYBH had three peaks (Fig. 8c). The first highest value of complexity was observed at 0 m in the shallow layer/oxic layer, whereas the microbial community showed the second highest complexity value at 110 m in the intermediate layer/chemocline. The highest microbial interaction network complexity value was at 250 m in the deep/anoxic layer.

## DISCUSSION

### The acclimation of Symbiodiniaceae to an extreme environment may rely on archaea in blue hole

Almost all genera and clades of Symbiodiniaceae in the SYBH were identified (except for *Effrenium*), and the *Cladocopium* C1 subclade was a core microbial taxon found in all water layers, suggesting that many members of Symbiodiniaceae have developed strategies to adapt to lightless extreme environments with high concentrations of sulfide, CH_4_, and N_2_O. This adaptive pattern or strategy may be diverse and complex because Symbiodiniaceae contains abundant species and complex genomes with many redundant functional traits (66, 67). However, the alpha diversity of Symbiodiniaceae showed a substantially positive correlation with that of archaea, and they remained relatively stable in the extreme environments of the SYBH. This stable positive correlation was almost unaffected by fungi and bacteria or their synergistic effects in SYBH, suggesting that some Symbiodiniaceae species possess the capability to thrive in extreme habitats, potentially dependent on the collaborative support of the archaea community within SYBH. Symbiodiniaceae species are closely associated with bacteria (*Endozoicomonas* and *Ralstonia*) (68 – 70). Nevertheless, the association between Symbiodiniaceae and archaea remains unclear (71). However, Thaumarchaeota was able to participate in the carbon fixation process of the *Porites lutea* holobiont, the ecological function of which was partly similar to that of carbon-fixing Symbiodiniaceae (72, 73). Thus, there was a partial niche overlap between the two microorganisms. In Symbiodiniaceae, the processes of photosynthesis and carbon fixation can be limited by extreme conditions with a lack of light and high levels of anaerobic products in the SYBH. However, archaea that live in diverse extreme environments possess sufficient ability to respond to such environments (28, 72, 74), which potentially offers an ecological foundation for providing extra sources of carbon and nitrogen to specific species of Symbiodiniaceae. Archaea in the SYBH also exhibited carbohydrate metabolism, carbon fixation and metabolism, and nitrogen and sulfur metabolism and members of DPANN, which are dominated archaeal communities. This contributed to the α-diversity of archaea in the SYBH and their ability to live in marine extreme habitats that are filled with anaerobic products, in addition to showing symbiotic potential (28, 73, 75, 76). DPANN must rely on interactions with other archaea or microorganisms to obtain essential biomolecules, because most DPANN archaea share metabolic capabilities and limited genetic material (73). Therefore, diverse unclassified archaea taxa may provide key pathways of anaerobic product metabolism and carbon and nitrogen cycling to Symbiodiniaceae under lightless conditions and maintain energy acquisition and normal life activities of potential symbioses. Excluding photosynthesis, high expression levels of genes associated with carbonic anhydrase and ammonium transporters have been observed in *Cladocoplum*, whereas *Symbiodinium* was capable of fixing carbon and assimilating inorganic nitrogen. This process is required for activities, such as bicarbonate transport and the function of NAD-specific glutamate dehydrogenase (44), thus enhancing pathways regulating core metabolism and key genes involved in carbon and nitrogen cycling within Nanoarchaeota and Aenigmarchaeota (73).

### The distinct ecological characteristics and environmental response patterns among microorganism in SYBH

The environmental response patterns were different among Symbiodiniaceae, archaea, bacteria, and fungi of the microbial assembly in the SYBH. The correlation coefficient between environmental factors and macro-diversity of Symbiodiniaceae and archaea exhibited a greater degree of stability than that observed for bacteria and fungi. The alterations observed in the α- and β-diversities of Symbiodiniaceae in response to environmental factors within the SYBH exhibited a high level of concordance with the corresponding patterns observed in archaea. Furthermore, a notable positive correlation was detected between the Shannon index (H′) values of Symbiodiniaceae and archaea, implying a potential dependent relationship between these two groups. Symbiodiniaceae and archaea Chl *a* response patterns of were different. The value of Chl *a* has often been used to indicate phytoplankton biomass and nutrient concentration in the ocean (77 – 80). However, a negative correlation was observed only between NO_3_^−^ concentrations and the α-diversities of Symbiodiniaceae and archaea, which were not correlated with any other nutrient concentrations. We speculate that the increase in α-diversity of archaea may be closely associated with an increase in the species and density of phytoplankton, because the potentially symbiotic Nanoarchaeota was found to be the main α-diversity contributor in archaeal communities. Nanoarchaeota lacks genes for amino acids, nucleotides, lipids, or cofactor biosynthesis (75) and exhibit an obligate symbiotic way of life with diverse hosts in extreme environments (81, 82). The marine phytoplankton genome contains key genes that Nanoarchaeota lacks (44, 83), and high-density and diverse phytoplankton may act as potential hosts for symbiotic or parasitic archaea, thereby increasing the α-diversity of archaea. The 63 phytoplankton species that have been found in the SYBH belong to Bacillariophyta (*n* = 49), Pyrrophyta (*n* = 12), Chrysophyta (*n* = 1), and Cyanophyta (*n* = 1) (84). Nutrient concentration was the main environmental factor affecting the dissimilarity between Symbiodiniaceae and archaea communities. An increase in NH_4_^+^, PO_4_^3−^, and Chl *a* concentrations reduced the β-diversity of Symbiodiniaceae, suggesting that an increase in metabolizable nutrient products may improve the stability and environmental tolerance of Symbiodiniaceae communities. Symbiodiniaceae promote growth and survival by obtaining NH_4_^+^ and PO_4_^3−^ from cnidarian hosts (66, 85, 86).

There was an increase in bacterial α-diversity and decrease in bacterial β-diversity in response to distinct environmental factors, which indicated that increased α-diversity may improve the stability of the bacterial community, thereby assisting bacteria to rapidly adapt to the extreme environments of the SYBH. Temperature was the most important factor for bacterial α- and β-diversities; however, the ocean regions or water layers (mesopelagic) with high bacterial α-diversity generally have low species and functional β-diversity and high functional richness (87). The sea surface bacterial community displayed the highest Shannon value in the eastern Indian Ocean at 25–35° S, and the dissimilarity degree of the bacterial community was the lowest in the same ocean area (88). Observations of bacterial community variability in the SYBH also supported microbial Anna Karenina effects, which suggested that low dispersion of the microbiome is associated with strong environmental tolerance and community stability under stress. This hypothesis has been verified in animal microbiomes (89 – 92). However, the changing rules of α-diversity for the fungal community were consistent with those of β-diversity, suggesting that the environmental response pattern of fungi in the SYBH was entirely different from that of bacteria. Within the interaction network in the SYBH, fungi, the dominant microbial group, participated in both synergistic and antagonistic interactions with all other microorganisms and showed abundant ecological functions. Microbial interactions are important drivers of environmental response patterns and ecological functions of fungal community structures (64, 93, 94), especially prokaryote interactions. The fungal community in the SYBH was dominated by Chytridiomycota, some members of which have displayed hyperparasitic abilities; Rozellomycota may be a hyperparasite of Chytridiomycota (95). Mycoparasitism of fungi increased the number of interactions between fungi and the dissimilarity of the fungal community. An increased number of members displaying parasitism or mycoparasitism have similar lifestyles and ecological niches (96) and share the same planktonic or fungal host species, leading to diverse fungal species combinations (64, 97, 98). Therefore, the increased dissimilarity of fungal communities is closely associated with high flexibility, which may contribute to more interactions within the fungal community itself or with other microorganisms, thereby promoting the environmental response of fungi in the SYBH. The variation patterns of fungal communities in SYBH were consistent with the anti-Anna Karenina effects. Increased bacterial α-diversity reduced fungal α-diversity, and this may be closely associated with the antagonism between bacteria and fungi, which are widespread owing to direct competition for resources (64, 94, 99). The SYBH is located in layers having dead plankton as the primary source of food, which also act as a carbon source for the SYBH (34), possibly causing the antagonistic relationship between the fungi and bacteria in the SYBH.

### Insight into the adaptability of core microbiome to respond to extreme environment in the SYBH

The *Cladocoplum* C1 subclade dominated the Symbiodiniaceae community and was a member of the core microbiome homogenized across all water layers of the SYBH, suggesting that the C1 subclade shows potential for adapting to dark, cold, oxygen-deficient, turbid, and other extreme environments. C1 subclade has high photosynthetic efficiency (100), which established symbioses with corals in cold marginal reefs and acclimated to environments with seasonal low temperatures, low DO, high turbidity, and eutrophication (101 – 104). Symbiodiniaceae communities of corals in the northern South China Sea have been dominated by the C1 subclade (14.2%–83.8%), the relative abundance of which showed a substantially negative correlation with sea surface temperature and a positive correlation with Chl *a* and Turb (65). *Cladocopium* is a functionally diverse and ecologically abundant genus of Symbiodiniaceae (43), and the C1 subclade may be the present ancestors of *Cladocopium* (105, 106). Thus, the C1 subclade may have evolutionarily diverged earlier than the other types of *Cladocopium* and experienced more local and global climate events, thereby providing a basis for some members of the C1 subclade to acquire strong tolerance to extreme environments.

*Alteromonas* was able to survive in aerobic, minimal oxygen, and anaerobic environments, demonstrating its ability to adapt to high turbidity and sulfides. *Alteromonas* is widely distributed in tropical and temperate oceans (107) and thrives in the Eastern Tropical South Pacific-oxygen minimum zones (ETNP-OMZ) (108), because many species of *Alteromonas* are particle-associated microaerophilic bacteria (62). *Alteromonas* also occurs in dark deep oceans; *Alteromonas macleodii* has been found in the Mediterranean and North Atlantic at depths of 0–680 m (109). However, *Alteromonas*, which is rarely found in completely anaerobic and sulfide-filled environments, prefers to live in ocean environments with sufficient phytoplankton-associated organic matter and nitrate reduction (62, 110). The relative abundance of *Alteromonas* sp. increased sharply at 200 and 250 m, suggesting that *Alteromonas* may have the ability to acclimate to oxygen-free extreme environments with high concentrations of turbidity and sulfide. *Ralstonia* is rarely found in the ocean, which is a beneficial bacterium that inhabits coral holobionts and maintains an endosymbiotic relationship with Symbiodiniaceae (45, 70). Although this study suggests that *Ralstonia* sp. shows strong adaptability in the SYBH, the relative abundance of *Ralstonia* sp. was highest on the sea surface (0 m) of oligotrophic coral reef water regions. *Ralstonia*, which is a genus of heterotrophic bacteria, displays recurrent “bloom” characteristics in the oligotrophic Eastern Mediterranean Sea and is highly abundant at intermediate depths in the summer (111). These results indicated that *Ralstonia* exhibits a broad extreme environmental acclimatization threshold and prefers to live in surface and intermediate oligotrophic sea areas.

Only two core archaeal ASVs belonging to Nanoarchaeota have been identified, and these also dominated the archaeal community in the SYBH. Nanoarchaeota prefer to live in aquatic habitats and are highly abundant in microbial communities in extreme environments (23, 28, 112). However, this environmental tolerance may rely on the symbioses between Nanoarchaeota and other archaea that carry key genes for sulfur metabolism (82). Thus, the environmental adaptability of Nanoarchaeota may change when they are distributed in the SYBH without compatible symbiotic archaea or with only dinoflagellates as potential symbiotic partners (73, 113). This study found that the relative abundance of core archaeal ASVs of Nanoarchaeota showed a peak in the chemocline (from 80 to 110 m), suggesting that some members of Nanoarchaeota not only survive in extreme environments but also prefer to live in ocean water layers with decaying organic matter that release oxidizing chemicals and reduced species together (36). Nanoarchaeota carries genes only related to glycolysis and gluconeogenesis pathways associated with carbon metabolism, which may lead to an intermediate process of organic matter degradation in the chemocline. However, there were no genes related to nitrogen and sulfur pathways in the Nanoarchaeota genome (73), leading to a sharp decrease in the relative abundance of core archaeal ASVs in the deep-water layers of the SYBH.

The number of core fungi taxa was higher than that of other microorganisms, and taxa were predominant in the fungal community of the SYBH, excluding *M. tassiana*. This suggests that the species constituting the core fungi assembly may have a wide environmental tolerance threshold. *M. tassiana* is homogenized in all water layers in SYBH, but this genus is also widely distributed in the deep sea and has been frequently detected in sediments in the dark and deep sea regions subjected to high pressure in the Pacific Ocean (25). The relative abundance of the *Recurvomyces* sp. in the intermediate and deep-water layers of the SYBH was higher than that in the shallow layers, which may be due to low pH and DO environments. *Recurvomyces* belongs to the Teratosphaeriaceae family, and some species of this family have survived in acid mine drainage situations (AMG; pH 2–8) (114). Thus, a decrease in pH in the 50-m water layers of the SYBH may lead to the enrichment of *Recurvomyces* sp. *Penicillium* often occur in extreme environments (64), the deep sea (115, 116), polar system (117), nuclear reactors (118), and international space stations (119 – 121). *P. cosmopolitanum* were identified in all water layers of the SYBH. Although *Penicillium* has a strong tolerance to diverse extreme environments, it prefers to live in surface oligotrophic sea areas, as demonstrated by its highest abundance in the 0-m water layer of the SYBH. Accordingly, the core microbiome survived in all water layers of the SYBH with varied environmental gradients, suggesting that the core microbiome has a wide environmental tolerance threshold and strong adaptability in response to diverse extreme environments.

### Microorganism has distinct ecological niches and biogeochemical functions in extreme environment of the SYBH

In oxic layers, the carbon metabolism profiles of bacteria and archaea displayed high abundance in the 20- and 50-m water layers, respectively. The shallow ocean in the SYBH shows extensive degradation of DOC and an increase in modern dissolved inorganic carbon concentrations (34, 36), which is due to not only a rapid decline in DO and accumulation of H_2_S but also the carbon metabolism of bacteria and archaea (59). Although the carbon metabolism process in the chemocline is dominated by the bacterial community, both archaea and bacteria are involved in carbon fixation and metabolism in deep anoxic layers. The fungal function group of animal pathogen-plant pathogen-undefined saprotrophs was enriched in the lower boundary of the chemocline, suggesting that the fungi were the main contributors to the degradation of particulate organic matter in the chemocline. Phytoplankton has the highest biomass in the 50–85-m water layers located in the upper boundary of the chemocline (37, 84), and this may be closely associated with the enrichment of fungal function groups of unclassified saprotrophs in the 85-m water layer. These fungi may utilize organic matter produced by living phytoplankton (64, 122). Nevertheless, fine particulate organic matter produced by the decomposition of phytoplankton and polysaccharides may be enriched in the lower boundary of the chemocline because of the strongly stratified system and wake water exchange between the chemocline and the deep anoxic layer (36, 58), which may provide suitable conditions for the enrichment and organic matter decomposition of saprotrophic fungi. However, the concentration of POC in the 110-m layer did not increase, suggesting that the decomposition rate of organic matter for microorganisms may be higher than its accumulation rate in the chemocline. The active fungal community in the lower boundary of the chemocline was also supported by animal-plant pathogens and the maximal complexity of the microbial interaction network that was driven by the fungal community.

Regarding nitrogen metabolism in the SYBH, nitrate reduction and nitrogen metabolism function profiles are enriched by bacterial and archaeal communities in the 95-m layer of the chemocline, which is closely associated with an increase in nutrient concentrations (36, 59, 62). Although functional genes of aerobic ammonium oxidation and complete denitrification are enriched in microbial communities in the chemocline (90–100 m) (62), the abundance of these genes may be mainly attributed to bacteria rather than to archaea. The abundance of 16S rRNA genes of bacteria (1.0 × 10^4^–5.0 × 10^7^) was higher than that of archaea (1.0 × 10^4^–1.0 × 10^5^) in the chemocline (80–110 m) (59). Additional nitrogen, especially NH_4_^+^, was also utilized by bacteria in the lower boundary of the deep anoxic layers (36), because the functional profile of nitrogen metabolism in the 250-m water layer was enriched only by the bacterial community (Fig. 6a).

Although sulfides from the chemocline or deep anoxic layers are rapidly oxidized in oxic layers (above 80 m; Fig. 1d), due to being catalyzed by dissolved trace metals (20, 36), the short duration of sulfide oxidation may provide survival conditions for archaea that are able to metabolize sulfides. This study found that the sulfur metabolism trait was enriched by the archaeal community inhabiting layers 50 m above the chemocline (Fig. 6b). Thus, archaea may be involved in the decomposition of sulfide that spills from the chemocline to the oxygen layer in the SYBH. The relative abundance of Desulfobacterota increased from 0.14% at 80 m to 5.9% at 100 m, and the functional profile of the sulfur relay system was enriched by the bacterial community at 95 m, which suggested that abundant bacterial taxa were involved in each step of sulfur cycling in the chemocline. The lower boundary of the chemocline was lightless and contained both oxygen and sulfide (36), which may have led to a high abundance of functional traits linked to dark oxidation of sulfur at 110 m, the profiles of which may have been contributed by ε-proteobacteria, which carry the highest number of genes that regulate sulfur-oxidizing multienzyme complex quinone-oxidoreductase, in the 110-m water layers (62). In the deep anoxic layer, functional profiles of the sulfur relay system were enriched by the archaeal community in the 150-m layers, indicating that the archaeal community is more competitive in sulfide metabolism than the bacterial community. However, the bacterial community showed enrichment characteristics of sulfur metabolism traits at 250 m, indicating that bacteria dominated the sulfur cycling process at the lower boundary of the deep layers of the SYBH. These two water layers showed active sulfur metabolism, as suggested by geochemical research (34). Therefore, archaea and bacteria with sulfur metabolism ability may have carved out distinct niches in the deep extreme environments of the SYBH.

Collectively, bacteria and archaea are involved in carbon, nitrogen, and sulfur cycles, while fungi play an important role in the microbial metabolism of carbon. These microbes have distinct ecological niches and biogeochemical functions in the extreme environment of the SYBH.

### Conclusions

Six genera and two clades of the Symbiodiniaceae were identified and widely distributed in the chemocline and deep anoxic layers of the world’s deepest blue hole. There was a substantially positive correlation between the α-diversities of Symbiodiniaceae and archaea. This improves our understanding of the adaptive threshold of Symbiodiniaceae and indicates the potential reliance of Symbiodiniaceae on archaea for acclimating to extreme environments, as a result of a partial niche overlap. There was a notable negative association between the α- and β-diversities of the bacterial community, suggesting that the change rule of the bacterial community was consistent with the Anna Karenina effects. However, the community structure variation of fungi was different from that of bacteria, which may be explained by the anti-Anna Karenina effects. The core microbiome in the SYBH comprised nine microbial taxa in *Cladocopium* sp., γ-proteobacteria, Nanoarchaeota, and Ascomycota, suggesting that they possess strong tolerance and adaptability to sharp environmental gradient variations, which may be associated with evolution, acclimatization, and symbiosis. Moreover, the ecological profiles of the microbiome showed significant enrichment characteristics among distinct water layers, wherein fungi played a key role in carbon metabolism, while bacteria and archaea participated in the biogeochemical cycle processes of carbon, nitrogen, and sulfur. Thus, these microbes have distinct ecological niches and biogeochemical functions in the extreme environment of the SYBH. This study provides insights into the community dynamics, tolerance threshold, and response pattern of the microbiome in the SYBH, thereby enhancing our understanding of the evolution and ecology of diverse microorganisms in extreme hydrospheric environments.

## MATERIALS AND METHODS

### Sample collection and environmental parameter measurements

A total of 56 seawater samples (10 L/sample) were collected in 14 depths across oxic, chemocline, and anoxic layers (0, 10, 20, 50, 80, 85, 90, 95, 100, 110, 120, 150, 200, and 250 m; four samples per depth) at the SYBH in June 2021 (Fig. 1). The seawater sample collection from the oxic and chemocline layers was conducted using a Conductivity Temperature Depth system 12-bottle rosette sampler (General Oceanics, USA), and from the deep anoxic layers (150, 200, and 250 m), seawater sample collection was conducted using an ROV equipped with GO-Flo bottles (General Oceanics). For microbiome analysis, the 5 L of seawater samples were pre-filtered through a 50 µm mixed cellulose esters membrane (Millipore, Billerica, USA), and large particles and organisms (e.g., cnidarian planula larvae) were removed. Subsequently, a 0.22-µm polycarbonate membrane (Millipore) was applied to collect cells of Symbiodiniaceae, bacteria, archaea, and fungi in seawater from distinct water layers. All polycarbonate membranes were transferred directly in 5-mL cryotubes, which were stored using liquid nitrogen until DNA extraction.

To evaluate the variation of environmental parameter and their association with microorganism, temperature (℃), salinity (PSU), DO (mg/L), pH, Turb (FNU), and Chl *a* (μg/L) were measured by conductivity temperature depth at the same time as that of water sample collection. The other 5 L of seawater samples in each layers was immediately filtered through a 0.45-µm mixed cellulose esters membrane; consequently filtrate and filter membranes were also stored at −80℃ for nutrient and SPM (mg/L) tests, respectively. NO_3_^−^ (μmol/L), NO_2_^−^ (μmol/L), NH_4_^+^ (μmol/L), SiO_3_^2−^ (μmol/L), and PO_4_^3−^ (μmol/L) were determined using an QuAAtro auto-continuous flow analyzer (SEAL, Germany). The concentration of biogenic gases (N_2_O, nmol/L; CH_4_, nmol/L), sulfide (μg/L), DOC (μmol/L), and POC (μmol/L) were determined as per previous studies (34, 36, 59).

### Microbiome DNA extraction, polymerase chain reaction amplification, and Illumina sequencing

The total microbiome DNA of all collection water layers was extracted using the Fast DNA Spin Kit for soil (MP Biomedicals, France), according to the manufacturer’s protocol. The concentration and purity of the microbiome DNA were assessed utilizing a NanoDrop2000 ultraviolet spectrophotometer (Thermo Fisher Scientific, MA, USA), and only high-quality DNA was selected as the template for PCR amplification. The primers ITSintfor2 (5′-GATTGCAGAACTCCGTG-3′) (123) and *ITS2*-reverse (5′-GGGATCCATATGCTTAAGTTCAGCGGGT-3′) (124) were used to conduct PCR amplification of *ITS2* from the Symbiodiniaceae rDNA. The V3–V4 region of the 16S rRNA gene of bacteria was amplified using primers 338F (5′-ACTCCTACGGGAGGCAGCAG-3′) and 806R (5′-GGACTACHVGGGTWTCTAAT-3′) (125). The V4–V5 hypervariable region of the archaeal 16S rRNA gene was amplified using primers 524F10extF (5′-TGYCAGCCGCCGCGGTAA-3′) and Arch958RmodR (5′-YCCGGCGTTGAVTCCAATT-3′) (126), and the fungal *ITS2* was amplified with the primer pair ITS3F (5′-GCATCGATGAAGAACGCAGC-3′) and ITS4R (5′-TCCTCCGCTTATTGATATGC-3′) (127). PCR was performed with ~10 ng of DNA, 1.6 µL (5 µM) primer, 0.4 µL *Trans* Start Fastplu DNA Polymerase, 0.2 µL BSA, 4 µL 5× FastPfu Buffer, 2 µL of 2.5 mM dNTPs, and ddH_2_O at a total volume of 20 µL. PCR amplification was conducted on an ABI GeneAmp 9700 thermocycler with the following program: 3 min at 95°C, followed by 35 cycles of 95°C for 30 s, 55°C for 30 s, 72°C for 45 s, and a final extension at 72°C for 10 min. The PCR products were run on a 2% ultra-pure agarose gel and purified using a Qiagen Agarose Gel DNA Purification Kit (Qiagen, Hilden, Germany). The amplicons were sequenced on an Illumina MiSeq platform using the 2 × 300 bp mode based on standard protocols at Majorbio (Shanghai, China) after entry quality control and adapter ligation.

### Microbiome identification and bioinformatics processing

QIIME 2 framework (version 2018.8) was used to sequence bioinformatics analysis of sequences (128). The DADA2 pipeline in R statistical software (version 4.2.1) was used to remove low-quality reads and concatenate the four amplicon reads (129), and qualified reads were rarefied to an equal sequencing depth (reads per sample: Symbiodiniaceae, 17,472; bacteria, 18,126; archaea, 7,665; and fungi, 34,251). After quality control, the reads were clustered into ASVs using the DADA2 algorithm. For Symbiodiniaceae *ITS2* data set analysis, the quality-filtered reads were aligned to the *ITS2* database using BLASTN, and the parameters were set following the pipeline detailed in previous studies (45, 65). To accommodate the use of *ITS2* as a multi-copy molecular marker, sequence-based *ITS2* analysis was used to identify dominant Symbiodiniaceae sub-clades, and the presence of *ITS2* sequences at a minimum cut-off of >5% in at least 1 of 56 water layer samples indicated biologically relevant entities of Symbiodiniaceae (41, 45, 55, 65, 130, 131). ASVs were used to analyze ecological indexes (α-diversity and β-diversity) of Symbiodiniaceae. However, not all ASVs that were clustered by *ITS2* reads belonged to Symbiodiniaceae, because the significance of individual base pair differences within the Symbiodiniaceae *ITS2* sequence was ambiguous (130, 131). Thus, after removing chimeras using the DADA2 pipeline (131, 132), the ASVs of *ITS2* were aligned to a non-redundant *ITS2* database using local BLASTN (65, 133). Subsequently, high-confidence ASVs corresponding to Symbiodiniaceae *ITS2* were identified and utilized for downstream analysis (45, 133). The taxonomic classification of bacterial 16S ASVs, archaeal 16S ASVs, and fungal ITS ASVs was conducted using the feature-classifier of QIIME 2 (128), which employs a Naïve-Bayes classifier. The classifier aligned the ASVs to the Silva v138/16S bacteria, Silva v138/16S archaea, and Unite 8.0/ITS fungi databases. A bootstrap confidence level of 0.7 was set for the classification process (134, 135). To enhance the quality of the ASV data, chimeric data and sequences originating from mitochondria, chloroplasts, and non-microbial sources were removed from the data sets. The downstream calculation of ecological indexes was performed using high-confidence ASVs of bacteria, archaea, and fungi.

### Statistical analyses

The α-diversity (Shannon H′) and β-diversity (Bray-Curtis dissimilarity) indices were calculated for the communities of Symbiodiniaceae, bacteria, archaea, and fungi in each sample using the high-confidence ASV data set. These calculations were performed using the Vegan package (2.6–4) in R (136). To assess the significance of differences in microbiome community, PERMANOVA was conducted using a Bray-Curtis dissimilarity matrix of high-confidence ASVs with 9,999 permutations. The PERMANOVA results were visualized by non-metric dimensional scaling generated by Bray-Curtis distance in R (Vegan package) (136). The α-diversity statistics were based on the relative abundances of ASV produced from the subsampled reads, and β-diversity calculations were performed based on subsampled and the total read abundances of ASV. Eighteen of the environmental factors in SYBH were correlated with α-diversity and β-diversity using GraphPad Prism 8 and Pearson’s correlation. The VPA was used to define the contributions of anaerobic (N_2_O, CH_4_, and sulfide), physical (depth, Temp, Sal, DO, pH, and Turb), and nutrient (NO_3_^−^, NO_2_^−^, NH_4_^+^, SiO_3_^2−^, PO_4_^3−^, DOC, POC, SPM, and Chl *a*) factors to the microbial community structure using the varpart function of the Vegan package (136). Redundancy of environmental variables was used to cluster variables using varclus in Hmisc R package (137).

Subsequently, QIIME 2 was used to identify the core microbiome (128). ASVs of Symbiodiniaceae, bacteria, archaea, and fungi consistently present in all depth groups were selected as conservative representative members of the core microbiome in SYBH (45). The GLM analysis was used to assess differences in relative abundance of core ASV with 1,000 bootstrap permutations, and the groups of depths was regarded as a fixed factor. The Student-Newman-Keuls (SNK) test was used for post hoc multiple comparisons of significant GLM test results. Moreover, phylogenetic investigation of communities by reconstruction of unobserved states 2 (PICRUST2) was applied to predict metagenomics functional content (KEGG-Pathway levels 1, 2, and 3) of dominant bacterial and archaeal community species (relative abundance > 1%) from the 16S rRNA marker gene (138). The “predict_metagenomes.py” was used to predict metagenome that the weighted nearest sequenced taxon index (weighted NSTI) was calculated for each sample (138). The annotation of prokaryotic taxa (FAPROTAX) was also applied to predict the ecological functions of bacterial and archaeal communities (139). Additionally, the prediction of fungal ecological function groups was performed using the FUNGuild tool (140). The Linear discriminant analysis Effect Size (LEfSe) method was used to identify shifts in the metagenome function abundance of microbial communities among distinct water layers (LDA threshold value = 2.0, *P* = 0.05) in the Galaxy web application (141).

A network modeling inference was applied to reveal the microbial interactions and key driver among Symbiodiniaceae, bacteria, archaea, and fungi in different water layers in SYBH with an extreme environment using the co-occurrence (CoNet) plugin for Cytoscape 3.9.1 (142, 143). Briefly, dominant Symbiodiniaceae *ITS2* sub-clades and other microbial ASVs as nodes were used to construct the molecular interaction network. The screening threshold was set as taxa present in at least two samples and having more than 30 reads. Two measures of correlations (Pearson and Spearman correlations), one measure of similarities (mutual information), and two measures of dissimilarities (Bray-Curtis and Kullback-Leibler dissimilarity) were applied to estimate pairwise correlations among these four microbial taxa in distinct water layers in SYBH. Initially, 1,000 positive and 1,000 negative edges were retrieved as thresholds for five measures, and 1,000 normalized permutations and 1,000 bootstrap scores were generated to mitigate the combinatorial bias. The measure-specific *P*-value was merged and calculated using Brown’s method (144). The Benjamini-Hochberg procedure was used to correct for multiple comparisons (145), and edges with merged *P*-values below 0.05 were retained. The co-occurrence interaction networks were visualized with Cytoscape 3.9.1, and the complexity of the interaction network was calculated as linkage density (links per ASV or subclade) among Symbiodiniaceae, bacteria, archaea, and fungi (146, 147). Additionally, the top 10 degrees of nodes in interaction have been defined as key drivers of the microbial interaction network in SYBH (41, 45). The correlations between network complexity of microbial community and depths were tested by linear fitting using Graph Pad Prism 8.

## Data Availability

The NGS raw read data set of these four amplicons has been submitted into the NCBI Sequence Read Archive database (Accession Numbers: PRJNA880990, PRJNA877068, PRJNA877090, and PRJNA898652).

## SUPPLEMENTAL MATERIAL

Supplemental tables spectrum.00531-23-s0001.xlsx Tables S1 to S4.

ASM does not own the copyrights to Supplemental Material that may be linked to, or accessed through, an article. The authors have granted ASM a non-exclusive, world-wide license to publish the Supplemental Material files. Please contact the corresponding author directly for reuse.
